# The Relationship between Adult Symptoms of Attention-Deficit/Hyperactivity Disorder and Criminogenic Cognitions

**DOI:** 10.3390/brainsci9060128

**Published:** 2019-06-02

**Authors:** Paul E. Engelhardt, Gavin Nobes, Sophie Pischedda

**Affiliations:** School of Psychology, University of East Anglia, Norwich NR77TJ, UK; g.nobes@uea.ac.uk (G.N.); s.pischedda@hotmail.co.uk (S.P.)

**Keywords:** criminogenic cognitions, criminal thinking, ADHD, inattention, hyperactivity/impulsivity

## Abstract

The relationship between ADHD—in particular hyperactivity—and criminal behavior is well documented. The current study investigated the role of criminogenic cognitions in the explanation of this relationship by examining which symptoms of ADHD are associated with criminogenic cognitions. Community-recruited adults (*N* = 192) completed self-report questionnaires for symptoms of ADHD and criminogenic cognitions. Symptoms of inattention were consistently and strongly related to criminogenic cognitions. In particular, inattention was significantly related to *cutoff, cognitive indolence,* and *discontinuity.* There was also evidence that impulsivity was positively related to criminogenic cognitions, and specifically, to the *power orientation* subscale. In contrast, and contrary to expectations, symptoms of hyperactivity were not related to criminogenic cognitions. These results indicate that in community-recruited adults, inattention rather than hyperactivity is related to criminogenic cognitions. We discuss the implications of these findings contrasting with those of previous studies that used forensic and clinical samples.

## 1. Introduction

A disproportionately high percentage of people with ADHD become involved with the criminal justice system. For example, a meta-analysis investigating ADHD and criminal behavior—including both minor offenses and crimes leading to incarceration—indicated a moderate-to-robust association [1]. Up to two-thirds of child or adolescent offenders, and half of adult offenders, show elevated symptoms of ADHD [2,3,4]. Some reports indicate that as many as half of young offenders [5] and 10%–15% of adult inmates [6] meet the diagnostic criteria for ADHD. Moreover, people with ADHD also tend to show higher rates of reoffending [4,7]. Longitudinal studies also suggest that individuals exhibiting ADHD in childhood are at higher risk of criminal involvement as adults compared with typically-developing individuals [8,9,10]. Gudjonsson, Sigurdsson, Young, Newton, and Peersen [11] found that over half of the prisoners they screened met retrospective diagnosis for childhood ADHD, and nearly two-thirds of these either met criteria (as adults) or were in partial remission [12]. Fletcher and Wolfe [13] and Moffitt [14] reported that individuals exhibiting ADHD symptoms at 5–12 years of age were significantly more likely than their peers to report criminal activities as young adults. 

There is a wide range of factors have been implicated in the ADHD-crime relationship (e.g., poor academic performance, truancy, poor parental management, defiance, and aggression) [1,15]. The most clearly established of these factors are associating with deviant peers and low self-control [1]. Investigation of these associated variables has contributed to the understanding of the etiology of ADHD, including genetic or biological and environmental risk factors (e.g., maternal smoking and low birth weight) that lead to or otherwise promote, factors such as low self-control and association with deviant peers. This research has also informed criminological theories (e.g., control models and Strain Theory) [16]. However, there remains little integration of these disparate theoretical perspectives in terms of how our understanding of ADHD might contribute to explanatory models of crime and delinquency [17]. Moreover, given that the associations have been already established [1], it is now possible for ADHD to be considered in treatment services (i.e., from youth interventions to rehabilitation and management of adult offenders). The current study contributes to these issues by identifying the *ADHD symptom clusters* that are most closely related to criminogenic cognitions in adult non-offenders.

There is some controversy concerning ADHD symptom clusters. For example, Babinski, Hartsough, and Lambert [18] found in a 9-year follow up study that childhood symptoms of hyperactivity/impulsivity, but not of inattention, were related to arrest records and self-reported crime [19], and that the effect of hyperactivity/impulsivity was over and above that predicted by conduct problems. In a similar study focusing on conduct problems and hyperactivity/impulsivity, Taylor, Chadwick, Heptinstall, and Danckaerts [20] also showed an independent effect for hyperactive/impulsive symptoms irrespective of the presence of conduct problems [4,21]. In contrast, Fletcher and Wolfe [13] reported that both inattention and hyperactivity/impulsivity contribute to the risk of criminal involvement. 

Thus, a large body of research indicates that the proportion of individuals with ADHD in the criminal justice system exceeds that in the general population. There is also substantial evidence to suggest that children with ADHD are more likely to engage in criminal activity throughout the course of development, including into adulthood. Moreover, the majority of the literature points toward impulsivity, and to a lesser extent hyperactivity, as the key factors in the ADHD–crime relationship [1]. However, the reasons for the link between ADHD and criminal behavior are less clear. One possible factor is *criminogenic cognitions*, that is, the problematic thought patterns (also known as criminal thinking) that precede criminal behavior. 

### 1.1. Criminal Thinking 

Walters [22] defined criminal thinking in terms of cognitive processes that induce a tendency to act in a criminal or anti-social manner. It has been identified as one of the best predictors of reoffending [23,24]. Criminals tend to have elevated levels of criminal thinking compared to those of non-criminals [25,26,27]. Moreover, there are differences depending on the type and severity of crimes committed—Walters [28] found higher criminogenic cognitions in maximum-security inmates compared with low-security inmates. Similarly, Mandracchia and Morgan [29] reported that inmates who received longer custodial sentences reported higher levels of criminogenic cognitions than individuals receiving shorter sentences. Yochelson and Samenow [30] were the first to establish a conceptual framework for understanding criminal cognitions. Based on interviews with incarcerated offenders, they argued that the criminogenic cognitions of criminals are pervasive and influence perceptions and actions in every aspect of life [31]. They proposed that to reduce or eliminate criminal behavior, it is first necessary to reduce or eliminate problematic thinking. 

Subsequently, Walters [28,32,33,34] developed a *lifestyle* model of crime, according to which criminal behavior is accompanied by a corresponding system of criminogenic cognitions, such as poor decision-making and blaming others for one’s own behavior. Walters argued that these cognitions maintain the criminal lifestyle, and that only by modifying them can we realistically hope to reduce criminal behavior. To measure criminal thinking quantitatively, Walters [28] developed the Psychological Inventory of Criminal Thinking Styles (PICTS), which consists of eight subscales (Table 1). Although the PICTS was designed to be used with offender samples, McCoy, Fremouw, Tyner, Clegg, Johansson-Love, and Strunk [35] demonstrated its ability to identify criminogenic cognitions in non-offenders and reported a significant correlation between PICTS scores and self-reported criminal behavior in typically-developing university students. 

### 1.2. The Current Study 

While the literature reviewed above has established a strong link between ADHD and criminal behaviour, relatively little is known about the reasons for the link [1]. According to both Yochelson and Samenow [30] and Walters [28], it is individuals’ thinking that largely determines how they behave. However, to date there has been very little research on the cognitive processes underlying (or supporting) criminal behavior that might help explain the ADHD-criminality link, beyond low self-control. The primary goal of the current study was to examine which symptoms of ADHD are related to criminogenic cognitions. We aimed to contribute both to the explanation of the link between ADHD and criminality, and to the understanding of the risk factors for criminogenic cognitions—and hence for criminal behavior—in general. 

Identification of these risk factors is likely to have important implications for the development of interventions (however, it is important to bear in mind that identification of cognitive mechanisms linking ADHD to criminality might only go so far in this regard, given that cognitive interventions produce limited impact in youth with ADHD (for an evidence-based treatment review, see the previous study [36]). If practitioners (e.g., forensic psychologists) are to mitigate criminal behavior, they must first understand the reasons for the maladaptive thinking patterns that underlie it [29,37]. The risk factors we focused on were the two symptom clusters of ADHD: inattention, and hyperactivity/impulsivity. Most previous research in this area has focused either on individuals diagnosed with ADHD, or on individuals within the criminal justice system who also present symptoms of ADHD. In this study, we took a different approach and investigated the relationship between symptoms of ADHD and criminogenic cognitions in typically-developing individuals. To avoid range restriction problems that are often characteristic of convenience samples (e.g., undergraduates), the majority of our participants were community-recruited adults. We recruited a large sample to further support generalizability and to ensure sufficient power so that parameter estimates (regression coefficients) would be stable. 

In the statistical analyses, we included age and gender in all models. With respect to gender, females tend to commit far fewer, and less severe and violent crimes, compared with males [38,39,40,41]. Therefore, we predicted that males would report higher levels of criminogenic cognitions compared to females. Age and criminal behavior have been shown to follow an inverse-U pattern [36,42], which peaks between 15 and 25 years of age. Because the current study tested adults (18 years and over), we predicted a negative relationship between age and criminogenic cognitions. Based on existing literature, we expected positive relationships between ADHD symptoms and criminogenic cognitions, and with respect to symptoms clusters, we expected a stronger relationship between hyperactivity/impulsivity and criminogenic cognitions than between inattention and criminogenic cognitions.

## 2. Methods

### 2.1. Participants

The sample consisted of 192 participants (age: 18–65 years, M = 35.95, SD = 14.49). Demographic information about the sample is provided in Table 2. Participants were recruited by a team of undergraduates using a variety of different methods (i.e., fliers posted on and off campus, email contacts of acquaintances, snowball sampling, and notices and requests on social media) to ensure as representative a sample as possible.

### 2.2. Materials

Each participant was given a pack of questionnaires including a demographic questionnaire, the Psychological Inventory of Criminal Thinking Styles (PICTS) [28,32], and the Conners’ Adult ADHD Rating Scale – Self-Report: Long Version (CAARS) [43].

### 2.3. Criminal Thinking Styles 

The PICTS consists of 80 questions. However, for the purposes of this study the two validity subscales were removed, leaving 64 items related to criminal thinking. Some questions implied that the individual had already committed serious crime. We re-worded these questions for use with a community-recruited (i.e., non-forensic) sample. Participants rated how strongly they agreed or disagreed with each item on a 4-point Likert scale ranging from 1 (disagree) to 4 (strongly agree). There are eight subscales (Table 1), and a “total” criminal thinking score was created by averaging participants’ responses to all 64 items. Higher levels of criminogenic cognitions represent higher PICTS scores. The PICTS has moderate-to-high internal consistency and test-retest reliability [28,44,45]. 

### 2.4. Conners’ Adult Rating Scale 

The CAARS consists of 66 items. This scale contains DSM-IV symptom indices for inattention and hyperactivity/impulsivity, as well as four factor-derived subscales: Inattention/memory problems (difficulties completing tasks, difficulties concentrating, forgetfulness, and disorganization), hyperactivity/restlessness (restlessness, fidgeting, and difficulty working for long periods on the same task), impulsivity/emotional lability (impulsivity, low frustration tolerance, quick/frequent mood changes, and being easily angered/irritated), and problems with self-concept (low self-esteem, low self-confidence, and generally poorer social interactions). Participants rated how strongly they agreed or disagreed with each item on a 4-point Likert scale ranging from 0 (not at all, never) to 3 (very much, very frequently). The CAARS has been found to have high internal consistency (α = 0.86 to 0.92) and test-retest reliability (*r* = 0.80 to 0.91) [46].

### 2.5. Procedure

Participants completed the questionnaires in their own time, and it was estimated to take each participant approximately 40 minutes. Once completed, questionnaires were returned to the experimenter and a debrief form was provided explaining the purpose of the study. Ethical clearance for the study was provided by the University of East Anglia Research Ethics Committee, and conformed to the protocols governing the use of human research participants outlined by the British Psychological Society. 

### 2.6. Data Preparation and Screening 

Data were first checked for outliers, which were defined (based on sample size) as values greater than four SDs from the mean. One PICTS score was more than five SDs from the mean, and so further scrutiny of this individual was undertaken. After ensuring that results were not due to any errors, we ran the main regression analyses twice; first with the participant in, and second, out of the dataset. Despite their high deviation from the mean, the case did not exert any substantive influence on the main patterns of findings. Therefore, we elected to retain the participant in the dataset. (Results of the main regression analyses excluding the potential outlier are presented in Table S1.) None of the other measures contained outliers. 

Transformations (square root, logarithm, and inverse) were then applied to skewed variables, which were defined as skew values exceeding twice the standard error. The transformations applied to each of the variables are reported in Table 3, and the transformations corrected skew to within three times the standard error. The raw scores from the CAARS questionnaire were tallied to produce a score for each subscale, which was then converted to a *T*-score. We did not apply transformations to *T*-scores for two reasons: first, they are age and gender standardized, and second, we wanted our findings to be comparable to the other studies in the literature. The descriptive statistics are presented in Table 3, and the bivariate correlations between variables are provided (for interested readers and future meta-analyses) in Table S2. Finally, we calculated split-half reliabilities for the CAARS and the PICTs across subscales using Spearman-Brown prophecy formula corrected coefficients; both demonstrated good reliability (CAARS = 0.78 and PICTs = 0.92). Further information about reliability is provided in Section C of the Supplementary Materials.

### 2.7. Data Analytic Plan

For the total PICTS scores, two backward multiple regressions were run: the first examined the factor-derived subscales (i.e., inattention/memory problems, hyperactivity/restlessness, impulsivity/emotional lability, and problems with self-concept), and the second examined the DSM-IV symptom indices (inattentive symptoms and hyperactive/impulsive symptoms). As mentioned previously, we also included age and gender. For the PICTS subscales, an additional set of backwards multiple regressions used each PICTS subscale as a criterion variable. Two steps were taken to avoid problems of multiple testing: first, as recommended by Stevens [47], only regression coefficients of ± 0.33 (i.e., twice the *r-*value for a significant bivariate correlation for *N* = ~200) or greater were interpreted; and second, we focused on results that patterned similarly (in terms of significance) for the factor-derived symptom domains and the DSM-IV indexes. In addition, for all regression analyses, the assumptions of regression (normal distribution of errors and homoscedasticity) were examined. 

## 3. Results

### 3.1. PICTS Total 

#### Factor-Derived Subscales

The first multiple regression examined whether the factor-derived subscales predicted total criminogenic cognitions. The overall model was significant *F*(4,187) = 52.13, *p* < 0.001. The *R*^2^ was 0.53, and age, gender, inattention/memory problems, and impulsivity/emotional lability were all retained as predictors (see Table 4). As predicted, higher age and being female were negatively related to criminogenic cognitions, and the factor-derived subscales were positively related to criminogenic cognitions. However, contrary to expectations, inattention/memory problems was more strongly associated with criminogenic cognitions than was impulsivity/emotional lability.

### 3.2. DSM-IV—Symptom Indices

A second (backwards) multiple regression using the two DSM-IV symptom indices showed that the overall model was significant *F*(4,187) = 44.75, *p* < 0.001. The *R*^2^ of the model was 0.49. Age, gender, inattentive symptoms, and hyperactive/impulsive symptoms were all retained (see Table 4). Similarly to the factor-derived subscales, inattention was three times more closely related to criminogenic cognitions than was hyperactivity/impulsivity.

### 3.3. PICTS Subscales 

Age was a consistent predictor of entitlement (i.e., entrenched thinking) and super-optimism, and impulsivity was a consistent predictor of power orientation, which is defined by aggression and manipulation (see Table 5). However, as with total criminogenic cognitions scores, inattention showed the strongest and most consistent results across the subscales. Inattention was consistently associated with three subscales: cutoff, cognitive indolence, and discontinuity. The latter two subscales closely follow symptoms of ADHD, insofar as both involve poor problem solving and inability to follow through on tasks and actions.

To investigate whether hyperactivity was related to criminogenic cognitions only at problematic levels, we ran a sub-group analysis including only the 37 participants with DSM-IV index *T*-scores > 60 (see Table S3) (*T*-scores of 60 or more are widely regarded as clinically impairing). The results for the sub-group differed from those of the full sample only in that neither factor-derived inattention/memory problems, nor DSM-IV index hyperactivity/impulsivity, was a significant predictor when regressed on to total criminogenic cognitions. We acknowledge that this analysis is likely underpowered given the number of predictor variables included in the model. However, when we examined *T-*scores > 55, the same pattern of results emerged, and the number of participants meeting this criterion was *N* = 47. Thus, examining even sub-impairment levels of ADHD symptoms showed little-to-no relationship between hyperactivity and criminogenic cognitions.

## 4. Discussion

The goal of this study was to investigate how symptoms of ADHD relate to criminogenic cognitions. Previous studies have tended to focus either on people diagnosed with ADHD or on individuals within the criminal justice system [13,48,49]. In contrast, in the current study, participants were community-recruited adults. Together, age, gender, and ADHD symptoms accounted for between half and two-thirds of the variance in criminogenic cognitions. As predicted, and consistent with previous research [28,38,50,51,52], older participants were less likely than younger participants, and women less likely than men, to endorse or report criminal thoughts. With regard to ADHD symptoms, impulsivity/emotional lability was also a significant predictor of total criminal thinking, and in particular, the *power orientation* subscale. Power orientation is related to power and control by aggressive and manipulative tendencies. This link is likely due to both involving, first, emotion (dys)regulation and lack of self-control, and second, strong reactions to frustration and perceived threats [53]. Consistent with this, a recent review [54] has implicated emotion dysregulation in social impairments and risky behaviors, as well as highlighted avenues for interventions for emotion dysregulation. 

However, for the DSM-IV indices, the standardized regression coefficient for inattention was three times larger than for hyperactivity/impulsivity. Similarly, the strongest ADHD subscale predictor of total criminal thinking was inattention/memory problems. This contrasts with our expectations, which were based on the findings of most previous research that hyperactivity/impulsivity would be a stronger predictor [1,13,55]. Inattention also significantly predicted three of the eight PICTS subscales, two of which—*cognitive indolence* (poor problem solving and critical thinking) and *discontinuity* (inability to follow through on thoughts and actions)—fit well with the diagnostic criteria of inattention and with theories of executive dysfunction in ADHD [56,57]. The other subscale (i.e., *cut off—*ignoring common psychological deterrents, such as anxiety and guilt) does not readily associate with symptoms of ADHD. However, these three PICTS subscales have been consistently identified in factor analysis studies on the PICTS [28,33,58], and those studies have labelled this trio of subscales *thoughtlessness and problem avoidance*, which again tends to fit well with descriptions of inattentive symptoms. 

Comparing the results of the two regressions, DSM-IV inattention was more closely related to criminogenic cognitions than was inattention/memory problems, which suggests that memory problems are not (or are only weakly) associated with criminogenic cognitions. Similarly, since impulsivity/emotional lability was more closely related to criminogenic cognitions than hyperactivity/impulsivity, and hyperactivity/restlessness did not predict criminogenic cognitions, it is likely that impulsivity is related to criminogenic cognitions, but that hyperactivity is not (or is only weakly) associated with criminogenic cognitions.

The strength of the relationship between inattention and criminogenic cognitions, and the absence of a clear relationship between hyperactive symptoms and criminogenic cognitions, are both surprising and somewhat counter-intuitive, because at least in children, problematic externalizing behavior is primarily due to the hyperactive/impulsive symptom domain. However, it is important to bear in mind that hyperactivity was not independently tested as a variable in this study. Thus, our conclusions regarding hyperactivity, at this point, should be interpreted with caution. The reason for these contrasting results might be that we tested adults, and there is some debate about how symptom patterns and subtypes remit over the course of development, particularly in adulthood [59]. In addition, whereas previous studies have tended to focus on individuals with clinically-impairing symptoms, the large majority of our participants were typically-developing and presented no problematic behaviors (A small number of participants had ADHD diagnoses or criminal convictions. However, they represent less than 7% of the sample, and we have no information regarding the type of crimes associated with the convictions.). However, this latter point was not supported by the analysis of participants with DSM-IV index *T*-scores > 60, although the analysis was slightly underpowered, as with the full sample inattention and impulsivity were significant predictors of criminogenic cognitions, and hyperactivity was not. Thus, our data showed no association between hyperactivity and criminogenic cognitions.

### 4.1. Implications and Future Research 

The divergent findings of this and previous research are likely to reflect the differing levels of criminal behavior shown by the samples. In contrast to participants in most previous studies, the community-recruited participants in the current study reported lower levels of criminality, and of course, were not incarcerated. This suggests that the combination of criminogenic cognitions and hyperactive symptoms (that is characteristic of previous studies’ samples) predicts criminality, whereas criminogenic cognitions in combination with inattention (as in the non-criminal participants in this study) does not. If correct, such an inference would have profound implications for our understanding of ADHD, criminogenic cognitions, and criminal behavior. There would also be important implications for policy and practice, primarily in the identification of individuals with ADHD symptoms who are at risk for criminality (because they show high levels of both criminogenic cognitions and hyperactivity), and of those who are not (because they show high levels of only one, or neither). In addition, interventions aimed at preventing or reducing criminal behavior by these at risk individuals should focus on addressing their criminogenic cognitions, ADHD symptoms, or both.

One approach to testing this possibility would be to compare rates of criminality among two groups of people with high levels of both criminogenic cognitions and ADHD. Those whose symptoms were of hyperactivity would be expected to engage in considerably more criminal behavior than those with symptoms of inattention. Another approach would be to conduct interventions designed to reduce criminal behavior by reducing criminogenic cognitions. We would predict that these interventions would be more successful when the primary diagnosis was of hyperactivity/impulsivity rather than inattention. 

### 4.2. Limitations 

Previous research has indicated that ADHD symptoms are a unique predictor over and above conduct problems. Unfortunately, in this study we were unable to collect assessments of conduct problems, and so we are not in a position to comment on how much of the variance in our ADHD-on-criminogenic cognition results may be shared with conduct problems, and how much variance is unique to ADHD [58]. A second limitation concerns the cross-sectional and correlational nature of the design. Future work is necessary to understand how criminogenic cognitions and their relationship to ADHD changes over the course of development. Third, we have relied exclusively on self-report for diagnostic symptoms. Much research has shown that adults tend to under-report symptoms of ADHD. Ideally, assessments would be collected from peer-informants, and if ADHD were suspected, a structured clinical interview for Axis I Disorders would be conducted. Finally, we did not include the PICTs validity scales, and so, despite the results of our outlier analysis, we cannot assess whether any participants adopted problematic response strategies. 

## 5. Conclusions 

These findings indicate that, as well as age and gender, criminogenic cognitions in community-recruited adults are strongly related to inattention, moderately related to impulsivity and impulsivity/emotional lability, and not related to hyperactivity. Given that ADHD symptoms tend to remit over the course of development, we feel that these results are particularly important to understanding the relationship between adult symptoms of ADHD and criminogenic cognitions, and how the understanding of that relationship is important for understanding the relationship between criminogenic cognitions, ADHD, and criminal behavior.

## Figures and Tables

**Table 1:** Psychological Inventory of Criminal Thinking Styles (PICTS) subscales and descriptions (adapted from Walters, 1995) [28].

Name	Description
1. Mollification	Rationalizing norm violation by blaming the cause of behavior on external events
2. Cut off	Ignoring common psychological deterrents of crime such as anxiety and guilt
3. Entitlement	Feelings of ownership, feelings of being justified in immoral behavior and a misidentification of wants as needs
4. Power Orientation	Pursuit of power and control over others, often by aggression and manipulation
5. Sentimentality	Attempts at compensating for and justifying past actions by doing good deeds
6. Super Optimism	Believing one can continue behavior without negative consequences
7. Cognitive Indolence	Poor problem solving and a lack of critical thinking especially towards one’s own plan and ideas
8. Discontinuity	Disruption of thought and lack of consistency and inability to follow through on thoughts and action (i.e., good intentions but poor self-discipline)

**Table 2:** Sample characteristics.

	*N*	%
Gender		
Male	78	40.6
Female	114	59.4
ADHD diagnosis		
Yes	4	2.1
No	188	97.9
Criminal conviction		
Yes	9	4.7
No	183	95.3
Police caution		
Yes	24	12.5
No	168	87.5
Education level (highest achieved)		
None	8	4.2
GCSEs	58	30.2
A-Levels	63	32.8
Undergraduate Degree	39	20.3
Postgraduate Degree	19	9.9
PhD	5	2.6
Occupation		
Employed (full time)	92	48.2
Employed (part time)	35	18.3
Unemployed	11	5.8
Student	53	27.7

ADHD, Attention-Deficit/Hyperactivity Disorder.

**Table 3:** Descriptive statistics for the Conners’ Adult ADHD rating scale and the Psychological Inventory of Criminal Thinking Styles (*N* = 192).

Measure	Mean	SD	Minimum	Maximum	Skew	Kurtosis
**Conners’ ADHD rating scale**						
Inattention/memory problems ^a^	7.06	0.67	5.83	9.43	0.492	0.535
Hyperactive/restlessness	48.18	8.97	30.0	73.0	0.460	−0.303
Impulsive/emotion lability ^a^	6.90	0.74	5.66	8.94	0.555	−0.188
Problems with self-concept ^a^	6.93	0.68	5.83	8.83	0.604	−0.269
DSM-IV inattention	50.95	13.29	28.0	90.0	0.638	0.231
DSM-IV hyperactive/impulsive	48.14	10.94	29.0	88.0	0.711	0.478
**Psychological Inventory of Criminal Thinking Styles**						
Mollification ^b^	0.77	0.19	0.25	1.00	−0.377	−0.899
Cutoff ^c^	0.17	0.13	0.00	0.54	0.505	−0.404
Entitlement ^b^	0.81	0.17	0.25	1.00	−0.644	−0.266
Power orientation ^c^	0.17	0.13	0.00	0.57	0.516	−0.313
Sentimentality ^c^	0.23	0.10	0.00	0.53	0.296	−0.167
Super optimism ^b^	0.73	0.15	0.25	1.00	−0.450	−0.001
Cognitive indolence ^a^	1.32	0.20	1.00	1.87	0.347	−0.328
Discontinuity ^c^	0.21	0.15	0.00	0.56	0.212	−0.849
Total ^c^	0.18	0.09	0.01	0.46	0.481	−0.347

^a^ square root transformation; ^b^ inverse transformation; ^c^ logarithm transformation.

**Table 4:** Regression coefficients for retained predictors on total criminal thinking (*N* = 192).

Variable	B	SE (B)	β	*t*-Value
Regression 1, with factor-derived subscales				
Age	−0.002	0.000	−0.33	−6.48 **
Gender	−0.046	0.010	−0.23	−4.61 **
Inattention/memory problems	0.058	0.009	0.40	6.11 **
Impulsivity/emotional lability	0.030	0.009	0.23	3.45 **
Regression 2, with DSM-IV indices				
Age	−0.002	0.000	−0.24	−4.37 **
Gender	−0.023	0.011	−0.12	−2.20 *
Inattention	0.003	0.001	0.48	6.76 **
Hyperactivity/impulsivity	0.001	0.001	0.13	1.78 ^#^

** *p* < 0.01; * *p* < 0.05; ^#^ *p* < 0.08. Gender coded male = 0 and female = 1. B, unstandardized regression coefficient, SE(B), standard error of unstandardized regression coefficient, β, standardized regression coefficient, *t*-value is the t-value of each predictor.

**Table 5:** Regression model *R*^2^s and βs of retained predictors on PICTS subscales (*N* = 192).

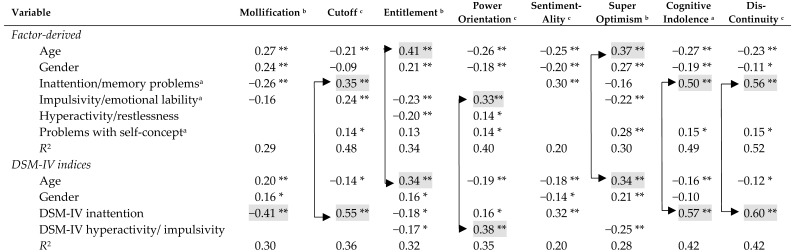

** *p* < 0.01; * *p* < 0.05; ^a^ square root transformation; ^b^ inverse transformation; ^c^ logarithm transformation. Gender coded 0 = male and 1 = female. Only shaded βs (> |0.33|) were interpreted.

## Supplementary Materials

The following are available online at https://www.mdpi.com/2076-3425/9/6/128/s1. Table S1: Results of the two main regression analyses following potential outlier exclusion (compare Table 4 main text). Table S2: Bivariate correlations between variables. Table S3: Regression coefficients for retained predictor variables on total criminal thinking for high ADHD scorers (*N* = 37).
