# Supplementary material for: The Relationship between Adult Symptoms of Attention-Deficit/Hyperactivity Disorder and Criminogenic Cognitions
Source: Brain Sci. 2019 Jun 2;9(6):128. doi: 10.3390/brainsci9060128 (PMC6627881; doi:10.3390/brainsci9060128)
Supplement: Supplementary file 1 [file brainsci-09-00128-s001.pdf]

Supplementary Materials: The Relationship between Adult Symptoms of Attention-Deficit/Hyperactivity Disorder and Criminogenic Cognitions

Paul E. Engelhardt, Gavin Nobes and Sophie Pischedda

Table S1. Results of the two main regression analyses following potential outlier exclusion (compare Table 4 main text).

Variable	B	SE (B)	β	*t*-value
Regression 1, with factor-derived subscales $F(4,186) = 52.13$, $p < 0.001$, $R^2 = 0.59$				
Age	−0.002	0.000	−0.31	−6.47 **
Gender	−0.050	0.009	−0.26	−5.52 **
Inattention/memory problems	0.056	0.009	0.40	6.54 **
Impulsivity/emotional lability	0.035	0.008	0.28	4.51 **
Regression 2, with DSM indices $F(4,186) = 54.68$, $p < 0.001$, $R^2 = 0.54$				
Age	−0.001	0.000	−0.22	−4.15 **
Gender	−0.025	0.010	−0.13	−2.63 **
Inattention	0.003	0.000	0.49	7.35 **
Hyperactivity/impulsivity	0.001	0.001	0.16	2.44 *

**, $p < 0.01$, *, $p < 0.05$, #, $p < 0.08$. Gender coded male = 0 and female = 1.

Table S2. Bivariate correlations between variables.

**Variable**	**1**	**2**	**3**	**4**	**5**	**6**	**7**	**8**	**9**	**10**	**11**	**12**	**13**	**14**	**15**	**16**	**17**
1. Age	-	−0.04	−0.09	−0.08	−0.18 *	−0.07	−0.29 **	−0.24 **	−0.39 **	0.31 **	−0.29 **	0.43 **	−0.33 **	−0.27 **	0.39 **	−0.32 **	−0.29 **
2. Gender		-	0.02	−0.10	0.03	−0.01	−0.15 *	−0.20 **	−0.23 **	0.22 **	−0.07	0.21 **	−0.18 *	−0.18 *	0.25 **	−0.17 *	−0.09
3. Inattention/memory			-	0.39 **	0.63 **	0.55 **	0.79 **	0.50 **	0.60 **	−0.37 **	0.59 **	−0.27 **	0.37 **	0.32 **	−0.17 *	0.61 **	0.66 **
4. Hyperactive/restlessness				-	0.51 **	0.17 *	0.47 **	0.78 **	0.38 **	−0.24 **	0.34 **	−0.32 **	0.38 **	0.23 **	−0.25 **	0.20 **	0.27 **
5. Impulsive/emotional lability					-	0.56 **	0.61 **	0.68 **	0.58 **	−0.36 **	0.57 **	−0.26 **	0.53 **	0.28 **	−0.22 **	0.47 **	0.53 **
6. Problems with self-concept						-	0.39 **	0.30 **	0.40 **	−0.22 **	0.48 **	−0.06	0.37 **	0.15 *	0.04	0.45 **	0.47 **
7. DSM–inattention							-	0.65 **	0.68 **	−0.49 **	0.59 **	−0.41 **	0.47 **	0.39 **	−0.32 **	0.63 **	0.64 **
8. DSM–hyperactive/impulsive								-	0.56 **	−0.36 **	0.45 **	−0.40 **	0.54 **	0.32 **	−0.37 **	0.37 **	0.45 **
9. PICTS–total									-	−0.77 **	0.81 **	−0.71 **	0.78 **	0.74 **	−0.64 **	0.81 **	0.78 **
10. Mollification										-	−0.53 **	0.67 **	−0.61 **	−0.66 **	0.57 **	−0.58 **	−0.53 **
11. Cutoff											-	−0.49 **	0.64 **	0.52 **	−0.44 **	0.70 **	0.74 **
12. Entitlement												-	−0.62 **	−0.60 **	0.67 **	−0.49 **	−0.40 **
13. Power orientation													-	0.55 **	−0.50 **	0.57 **	0.57 **
14. Sentimentality														-	−0.61 **	0.54 **	0.45 **
15. Super optimism															-	−0.40 **	−0.33 **
16. Cognitive indolence																-	0.74 **
17. Discontinuity																	-

*, $p < 0.05$, **, $p < 0.01$. Gender coded 0 = male and 1 = female.

Table S3. Regression coefficients for retained predictor variables on total criminal thinking for high ADHD scorers ($N = 37$).

Variable	B	SE (B)	β	t-value
Factor-derived subscales $F(3,33) = 18.91$, $p < 0.001$, $R^2 = 0.63$				
Age	−0.004	0.001	−0.54	−5.13 **
Gender	−0.093	0.019	−0.54	−4.80 **
Impulsivity/emotional lability	0.071	0.015	0.54	4.78 **
DSM indices $F(3,33) = 10.80$, $p < 0.001$, $R^2 = 0.50$				
Age	−0.004	0.001	−0.55	−4.41 **
Gender	−0.058	0.021	−0.33	−2.70 *
Inattention	0.003	0.001	0.35	2.78 **

**, $p < 0.01$, *, $p < 0.05$. Gender coded 0 = male and 1 = female.

Section C

CAARS.

The CAARS forms provide an inconsistency index, which can be used to identify participants who have adopted problematic response strategies. Because of this inconsistency index, we elected not to run reliability estimates on the individual questions of the CAARS data. Moreover, the CAARS forms are some of the most widely used screening forms for symptoms of ADHD and have been extensively tested (see Conners Manual 3rd Edition) for internal consistency, test-retest reliability, and inter-rater reliability. Across both Content Scales and DSM-IV Symptoms Scales, Cronbach's Alpha for internal consistency ranges from 0.85–0.91, test-retest reliability from 0.76–0.89, and inter-rater reliability from 0.70–0.84. With respect to the inconsistency index, below we have provided the frequencies from our sample. Typically, a score of more than 8 is cause for caution with regard to interpretation, especially for self-report. Our data showed four cases with an inconsistency score of 9, and perhaps more worryingly, one case with a score of 11, which clearly required follow up. Examination of T-scores (Content Scales and DSM-IV Symptoms Scales) for this participant revealed a range of 59–70, which is in the problematic range. This participant also reported an existing diagnosis of ADHD. In order to ensure that this particular individual did not substantially affect our results, we ran the two main regression analyses with this participant in and out of the dataset. The results in terms of the patterns of statistical significant and in fact the F-values and beta's were virtually identical. Therefore, we elected to retain this participant, despite the inconsistency of their CAARS scores.

Table S4. Table caption.

Inconsistency Index Guide

		Frequency	Percent	Valid Percent	Cumulative Percent
Valid	0.00	4	2.1	2.1	2.1
	1.00	8	4.2	4.2	6.3
	2.00	23	12.0	12.0	18.2
	3.00	43	22.4	22.4	40.6
	4.00	34	17.7	17.7	58.3
	5.00	28	14.6	14.6	72.9
	6.00	19	9.9	9.9	82.8
	7.00	19	9.9	9.9	92.7
	8.00	9	4.7	4.7	97.4
	9.00	4	2.1	2.1	99.5
	11.00	1	0.5	0.5	100.0
	Total	192	100.0	100.0	

PICTs

For the PICTS, we computed split-half reliabilities based on individual items. Because there were only eight questions for each subscale, we used Spearman-Brown prophecy formula corrected coefficients. The mean reliabilities were total PICTs = 0.89, and for the PICTS subscales, mollification = 0.64, cutoff = 0.74, entitlement = 0.49, power orientation = 0.66, sentimentality = 0.61, super optimism = 0.31, cognitive indolence = 0.55, and discontinuity = 0.80. Two of the subscales showed reliabilities falling below typically accepted thresholds (i.e., entitlement and super optimism). However, the published alphas in Walters' [33] meta-analysis ranged from 0.54–0.79, generally similar to those of the present study.
